# Retracted: Heme Oxygenase-2 Localizes to Mitochondria and Regulates Hypoxic Responses in Hepatocytes

**DOI:** 10.1155/2022/9801028

**Published:** 2022-05-11

**Authors:** Oxidative Medicine and Cellular Longevity

Oxidative Medicine and Cellular Longevity has retracted the article titled “Heme Oxygenase-2 Localizes to Mitochondria and Regulates Hypoxic Responses in Hepatocytes” [1] due to concerns with the data. In Figure 3(a) the Western blot lanes for HIF1- *α* appear to be identical to the first four lanes of the p-JNK blot in Figure 4(b).

The authors were contacted to clarify the duplication, however, due to the amount of time that has passed since publication, and the fact that the Western blots were not digitalised, they were unable to provide the raw data or a corrected image. Consequently, the article has been retracted with the agreement of the Editorial Board.

The authors agree with this retraction.
